# Crystal Structure and Biochemical Analysis of a Cytochrome P450 CYP101D5 from *Sphingomonas echinoides*

**DOI:** 10.3390/ijms232113317

**Published:** 2022-11-01

**Authors:** Pradeep Subedi, Hackwon Do, Jun Hyuck Lee, Tae-Jin Oh

**Affiliations:** 1 Department of Life Science and Biochemical Engineering, Graduate School, Sun Moon University, Asan 31460, Korea; 2 Research Unit of Cryogenic Novel Material, Korea Polar Research Institute, Incheon 21990, Korea; 3 Department of Polar Sciences, University of Science and Technology, Incheon 21990, Korea; 4 Genome-Based BioIT Convergence Institute, Asan 31460, Korea; 5 Department of Pharmaceutical Engineering and Biotechnology, Sun Moon University, Asan 31460, Korea

**Keywords:** α/β-ionone, crystal structure, cytochrome P450, X-ray crystallography

## Abstract

Cytochrome P450 enzymes (CYPs) are heme-containing enzymes that catalyze hydroxylation with a variety of biological molecules. Despite their diverse activity and substrates, the structures of CYPs are limited to a tertiary structure that is similar across all the enzymes. It has been presumed that CYPs overcome substrate selectivity with highly flexible loops and divergent sequences around the substrate entrance region. Here, we report the newly identified CYP101D5 from *Sphingomonas echinoides*. CYP101D5 catalyzes the hydroxylation of β-ionone and flavonoids, including naringenin and apigenin, and causes the dehydrogenation of α-ionone. A structural investigation and comparison with other CYP101 families indicated that spatial constraints at the substrate-recognition site originate from the B/C loop. Furthermore, charge distribution at the substrate binding site may be important for substrate selectivity and the preference for CYP101D5.

## 1. Introduction

Cytochrome P450 enzymes (CYPs) are heme-containing enzymes that catalyze the modifications of biologically active compounds, ranging from small molecules, such as camphor, to bulky fatty acids and carotenoids. These biological modifications, which mainly include monooxygenation, have been found to be beneficial for humans and other species [1]. In the human body, CYPs play an important role in the production and metabolism of hormones, such as estrogen, testosterone, cholesterol, and vitamin D [2,3,4], and metabolize various drugs (e.g., bilirubin) that are poisonous to humans [5]. Another notable feature of CYPs is their ability to transform medically useful polyketide peptides from polyketide synthases (PKSs), which may expand the diversity of small-molecule libraries for drug-discovery screening [6,7,8]. The pharmaceutical industry has focused on this exceptional property of CYPs to change the biological activity and physical properties of compounds. The modification of an inactivated C-H bond by CYPs may increase the solubility of compounds and convert them into active forms [9].

More than 1000 bacterial CYPs have been discovered and divided into approximately 150 families according to sequence similarity [8,10]. Bacterial CYPs have many advantages as biocatalysts. As indicated above, bacterial CYPs are composed of diverse homologues; even one bacterial genome can possess several homologues, indicating that bacterial CYPs can modify a vast range of substrates [8]. Unlike their eukaryotic counterparts, bacterial CYPs are soluble and can be obtained at a much higher yield [8]. Moreover, it is known that bacterial CYPs are more active than those from eukaryotes. However, bacterial CYPs have also shown disadvantages, such as the inaccurate region and/or stereo-selectivity of modifications and thermolabile properties [8,11].

Biochemical and structural studies have investigated and revealed that, although the primary protein structure for bacterial CYPs is not conserved, the secondary and tertiary structures of CYPs are similar to each other [12]. Sequence alignment studies have shown that the substrate recognition sites (SRSs) composed of five loops are neither conserved nor structurally rigid with various amino acid sequences [13,14,15,16]. The substrate selectivity or preference among diverse substrates of CYPs is thought to be overcome with highly flexible loops and divergent sequences around SRSs. However, the distinct rules for the structure-based substrate recognition of CYPs remain unclear.

In the CYP101 family, there are nine CYPs that are mostly from *Novosphingobium aromaticivorans* DSM12444 and *Sphingobium yanoikuyae* B2, except for CYP101A1 (P450cam), which is from *Pseudomonas putida*, and CYP101D3, which is from *Sphingomonas* sp. SKA58 [17,18]. CYP101D1, CYP101D2, and CYP101A1 catalyze the oxidation of camphor, while CYP101B1 and CYP101C1 use ionone as a substrate. Although intensive structural studies have been conducted, the selectivity between these two substrates of the CYP101 family has not been fully elucidated. It is, therefore, necessary to analyze diverse CYP structures to better understand the mechanisms underlying substrate recognition.

In this study, we report the biochemical characterization and crystal structure of CYP101D5 from *Sphingomonas echinoides* at a resolution of 3.2 Å. We explored the oxidation of terpenes and flavonoids by CYP101D5. The characterization of the products indicated that CYP101D5 hydroxylates and dehydrogenates various substrates. A structural analysis and comparison among the CYP101 subfamilies showed substrate-dependent residues and surface charge distribution at the substrate binding cavity. The results presented in this paper provide valuable structural information for selecting CYPs to modify or produce biologically important compounds.

## 2. Results and Discussion

### 2.1. Expression and Purification of CYP101D5

CYP101D5 was successfully expressed in soluble form in *E. coli* BL21 (DE3) cells. The sodium dodecyl sulfate-polyacrylamide gel electrophoresis (SDS-PAGE) analysis of the soluble fraction of CYP101D5 and redox partners, putidaredoxin (Pdx) and putidaredoxin reductase (PdR), showed a single and homogeneous band of purified proteins (Figure S1A). The theoretical molecular mass calculated for CYP101D5 was ~46 kDa. However, the SDS-PAGE analysis showed a band with a higher molecular weight (~53 kDa). The difference in molecular weight was due to the His-Tag/thrombine/T7-Tag sequence, which was fused to the N-terminal region of CYP101D5 and was translated along with it. The spectra of the oxidized form of CYP101D5 showed absorption at 418 nm, which is characteristic of CYP enzymes (Figure S1B). In addition, the amount of dithionite was reduced and the CO-bound form showed a peak at 448 nm, which is indicative of the native Fe^2+^CO complex form of CYPs [19].

### 2.2. Determination of P450 Activity and the Substrate Spectrum

As a preliminary study, we determined the redox partner for CYP101D5. The in vitro reactions were conducted using two heterologous redox partners, Pdx/PdR from *P. putida* and Fdx/FdR from spinach, and the chemical redox partners, hydrogen peroxide and (diacetoxyiodo) benzene. Since previously characterized CYP101 family members can hydroxylate β-ionone, we used this substrate to find the redox partner for bioconversion. The product peak was observed with β-ionone when Pdx/PdR was used as the redox partner, while slight or no activity was observed for the other redox partners. Pdx/PdR was, therefore, selected for the in vitro experiments.

**Figure 1:**
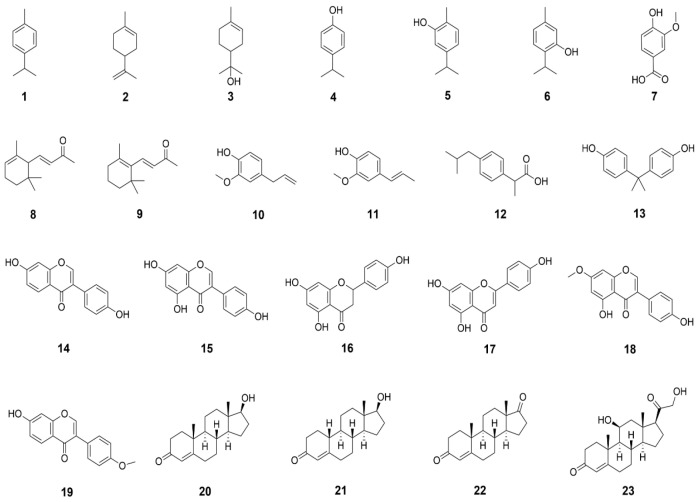
Substrate spectrum of CYP101D5. In vitro biotransformations were performed with purified CYP and Pdx/PdR as redox partners for 2 h using 23 substrates (1, cymene; 2, limonene; 3, α-terpineol; 4, 4-isopropylphenol; 5, carvacrol; 6, thymol; 7, vanillate; 8, α-ionone; 9, β-ionone; 10, eugenol; 11, isoeugenol; 12, ibuprofene; 13, bisphenol A; 14, daidzein; 15, genistein; 16, naringenin; 17, apigenin; 18, prunetin; 19, formononetin: 20, testosterone; 21, nandrolone; 22, androstenedione; 23, corticosterone).

To determine the substrate preference of CYP101D5, we screened the compounds that were identified as substrates of CYPs (Figure 1). The in vitro reaction of CYP101D5 was reconstituted with the purified redox partner Pdx/PdR and reduced nicotinamide adenine dinucleotide (NADH) as a cofactor. Each product of the monooxygenase reaction was analyzed using either gas chromatography–mass spectrometry (GC–MS) or high-performance liquid chromatography (HPLC) and liquid chromatography–mass spectrometry (LC–MS). The data showed that the biocatalyst was able to hydroxylate β-ionone, and the product was identified as 4-hydroxy β-ionone (Figure S2). A component identification was made based on mass spectral fragmentation. Previous studies have addressed the oxygenation of β-ionone by bacterial CYPs. CYPs from the CYP101 family, including CYP101B1 and CYP101C1, have been reported to produce the oxidized metabolite 4-hydroxy-β-ionone [20,21]. Similarly, other CYPs, including CYP102A1, CYP109B1, and CYP109D1, as well as P450 SU1, SU2, and SOY C, have also been shown to produce 4-hydroxy-β-ionone [22,23,24,25].

**Figure 2:**
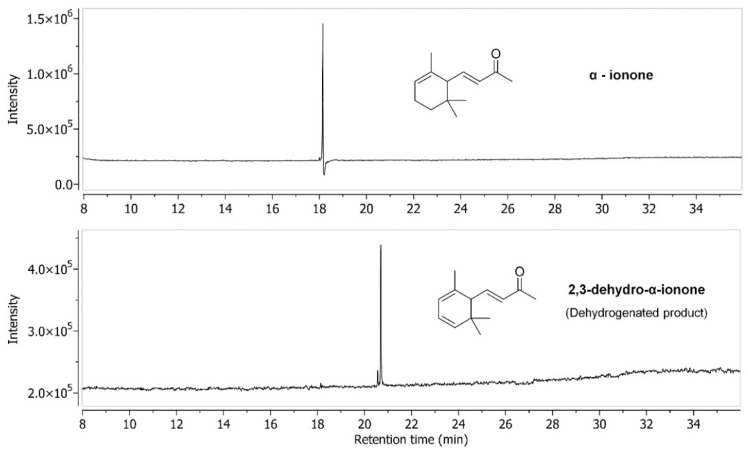
Gas chromatography analysis of the conversion of α-ionone by CYP101D5. The peak (t_RS_ = 18.18 min) is of the substrate and the product peak (t_RP_ = 20.68 min) is identified as 2,3-dehydro-α-ionone. The structures of both the substrate and product are shown. The mass spectra of both the substrate and product are presented in Figure S3.

The product for α-ionone was identified as the dehydrogenated product, 2,3-dehydro-α-ionone (Figure 2 and Figure S3). Dehydrogenation catalyzed by CYPs is not an uncommon reaction. There are several reports of P450-mediated dehydrogenation reactions [26]. Nevertheless, the dehydrogenation of α-ionone by CYP101D5 is an exceptional case, as the compound with a similar structure (β-ionone) produced the hydroxylated product. Many bacterial CYPs that are responsible for the hydroxylation of α-ionone into their corresponding mono-hydroxylated derivatives have been identified [20,24,27,28]. However, there are currently no reports of the enzymatic dehydrogenation of α-ionone. Yamazaki et al. first reported the microbial bioconversion of α-ionone to its corresponding dehydrogenated product, 2,3-dehydro-α-ionone. This study is the first to report the dehydrogenation of α-ionone catalyzed by the CYP101 family. We also attempted to purify the dehydrogenated product using large-scale bioconversion, but this was not possible due to its instability [29].

**Figure 3:**
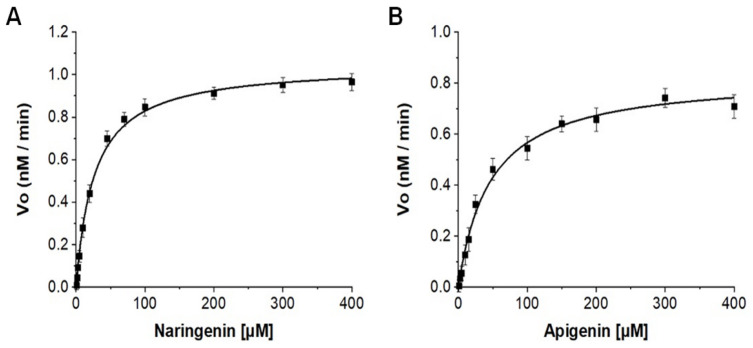
Determination of the kinetic parameters of naringenin and apigenin catalyzed by CYP101D5. The overall kinetic parameters were determined using CYP:Pdx:PdR at a ratio of 1:8:2. Hyperbolic fits of the hydroxylated products of naringenin (**A**) and apigenin (**B**) are shown. The bars on each point represent the standard deviation of the individual experiments.

The high-performance liquid chromatography–photodiode array (HPLC-PDA) analysis of the reaction mixture of naringenin and apigenin showed one product peak for each substrate (Figure S4). The LC–MS analysis showed one hydroxylated product (retention time for the peak (t_RN_)~11.42 min, (Figure S4A); calculated mass for the molecular formula of C_15_H_13_O_6_ for [M + H]^+^ *m*/z^+^~289.0711, for which the observed mass [M + H]^+^ *m*/*z*^+^~289.0704, λmax: 289 nm). Likewise, the reaction mixture of apigenin also showed one hydroxylated product (retention time for peak (t_RA_) ~11.25 min, (Figure S4B); calculated mass for the molecular formula of C_15_H_11_O_6_ for [M + H]^+^ *m*/*z*^+^~287.0555, for which the observed mass [M + H]^+^ *m*/*z*^+^~287.0557, λmax: 268 nm). The kinetic parameters of CYP101D5-catalyzed hydroxylation were calculated for the substrates naringenin and apigenin. The Michaelis–Menten constant (*Km*), catalytic rate constant, and coupling efficiency were calculated and are shown in Table 1 (Figure 3). The coupling efficiency of apigenin was lower than that of naringenin, indicating the loss of electrons from the cofactor NADH, which could have been due to the use of heterologous redox partners for catalysis.

We analyzed the purified hydroxylated product structures of naringenin and apigenin by ^1^H and ^13^C nuclear magnetic resonance (NMR) at 700 MHz in DMSO-*d*_6_ (Figure S5). The C-3′ of the product of naringenin showed an absence of the proton signal at δ = 6.75 ppm (m) and upfield shift of the carbon signal from δ = 115.63 ppm to δ = 145.20 ppm, accompanied by a downfield shift of the adjacent carbons C-4′ from δ = 158.20 ppm to δ = 145.72 ppm. We further identified the hydroxylated product as 3′,4′,5,7-tetrahydroxyflavanone (eriodictyol), which is a 3′-OH derivative of naringenin. The NMR spectral data were consistent with those in an earlier report [30,31]. The NMR analysis also showed that apigenin was hydroxylated at the 3′ position. Thus, the structure of the hydroxylated product of apigenin was identified as 5,7,3′,4′-tetrahydroxyflavone (luteolin). These data were strongly consistent with those in a previous report [32].

Although there have been reports of flavonoid hydroxylation by fungal and plant hydroxylases [33,34], flavonoid hydroxylation by bacterial CYPs has not been well studied. Bacterial hydroxylases have only been reported a few times, and they can only hydroxylate select flavonoids. CYP450 BM3 variants and CYP105D7 were able to hydroxylate select flavonoids, including naringenin [30,35]. A monooxygenase from *Saccharothrix espanaensis* (Sam5) hydroxylated the flavonoids [36]. The *E. coli* hydroxylase complex (HpaBC) also produced eriodyctiol through the 3′ hydroxylation of naringenin [37]. This study showed that, in addition to the hydroxylation of small molecules, CYP101D5 can also hydroxylate larger molecules, such as naringenin and apigenin. Additionally, the isoflavonoids daidzein, genistein, prunetin, and formononetin, which are similar in structure to the flavonoids naringenin and apigenin, showed no product formation. Isoflavonoids are a subclass of flavonoids and have a benzene ring linked to C-3 rather than the C-2 of benzopyran. Moreover, steroids with a similar but bulkier structure than that of flavonoids were not catalyzed.

### 2.3. Structure of CYP101D5

For further investigation, the three-dimensional structure of CYP101D5 was determined at a resolution of 3.2 Å using X-ray crystallography. The initial phase of the co-ordinate was obtained by molecular replacement (MOLREP) [38] using CYP101D1 from *N. aromaticivorans* (PDB code: 3LXH), which shares 55% sequence identity with CYP101D5 [18]. After the initial phasing, the iterative refinement using a combination of Coot [39] and Refmac5 [40] resulted in 26% and 35% of R_work_ and R_free_ values, respectively. Detailed information on the crystallographic and refinement statistics has been summarized in Table 2.

**Figure 4:**
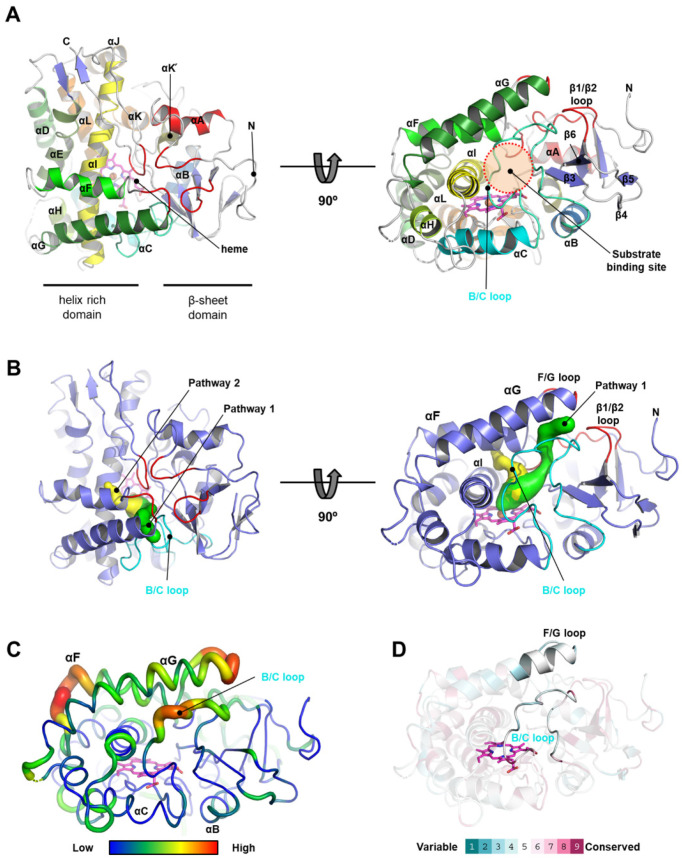
Overall structure and structural features of CYP101D5. (**A**) The overall structure of CYP101D5 is presented by a ribbon diagram with a top view (left) and a 90° rotated view (right). Loops close to the substrate recognition site and the B/C loop of CYPs are marked with red and cyan. The bound heme molecule is represented by a stick model in magenta. (**B**) The predicted substrate pathways for CYP101D5 were calculated by the program CAVER [42] and are presented in green and yellow. (**C**) B-factor distribution of CYP101D5 shown in a putty representation. Residues with a higher B-factor are presented in red. (**D**) Sequence variation of CYPs. The residues in a cartoon structure are colored according to their conservation grades using the nine-grade color-coding bar. The F/G and B/C loops are highlighted with no transparent presentation.

The final model of CYP101D5 contained two molecules in an asymmetric unit, and the two chains were similar to each other, as indicated by 0.620 of Cα RMSD. Minor differences between the two monomers were observed in the loop regions (residues 44–49 and 87–91). Since chain A showed greater electron density around the loop regions compared to chain B, we decided to use chain A to further analyze and describe the structure. The overall structure of CYP101D5 showed a typical CYP family fold with 13 α-helices (αA–αL, including αK՛), 8 β-strands (β1–β8), and connecting loops [41]. Like other CYP structures, CYP101D5 comprised a triangular shape and can be divided into two regions: the helix-rich domain consisting of αC, αD, αE, αF, αG, αH, αI, αJ, αK, and αL, and the β-sheet domain with three sets of antiparallel β strands, including β1 and β2, β4 and β5, and β6 and β7 (Figure 4A). The heme molecule was located in the central region between the two domains and residues Tyr72 from αB; His105, Arg109, Val112, and Leu116 from αC; Ala251 and The255 from αI; Val298 and Ala300 from the αK–β4 loop; Arg302 from β4; and Thr352, Phe353, His358, Ala361, and Gly362 from the αK’–αL loop, which are mainly involved in the interaction with the heme molecule by a combination of hydrophobic and hydrogen bonding and salt bridges (Figure S6).

### 2.4. Active Site and Substrate Access Channel of CYP101D5

The active site of CYP101D5 was located at the bottom of the inside of the funnel-like cavity composed of five loops, including the F/G and B/C loops on top of the heme molecule (Figure 4A). Among these loops, the long B/C loop largely contributed to the formation of the substrate binding cavity (Figure 4B). Previous studies on the CYP101 family have revealed that the substrate access channels of the CYP101 family are relatively narrow or buried by the surrounding residues. Wade et al. proposed three possible pathways (pw1, pw2, and pw3) and identified a common pathway using random expulsion molecular dynamics (REMD) and thermal motion pathway (TMP) analyses [43]. The active site of CYP101D5 was also isolated from the solvent-accessible region and did not show a clear route for the substrate. To identify the substrate access channel, we computed the pathway using the structure of CYP101D5 with the heme molecule (Figure 4B). The analysis indicated that CYP101D5 has two possible pathways. Pathway 1 starts from the heme plane and passes the edge of αG, the F/G loop, and B/C loop, which is close to the common pathway known as pw1 to the CYPs in Wade et al., 2004 [43]. The second pathway, named pathway 2, passes by the αI and extends to the triangular region composed of αF, αI, and a loop between αI and β8, which is similar to pw3 (Figure 4B). To further understand the substrate pathways, we analyzed the B-factor distribution of each residue [44]. The E/F, F/G, and B/C loops exhibited the highest B-factor values throughout the entire enzyme, indicating the vibrational motion of the residues (Figure 4C). Given that the turn regions of the F/G and B/C loops showed open conformations in the absence of substrates or products, while closed conformations were presented upon a substrate or the product binding of CYPs, such as the CYP105 and CYP101 families [45,46], pathway 1 is most likely the substrate access channel for CYP101D5 and changes its conformation during enzymatic catalysis.

### 2.5. Sequence Comparison of CYP101D5 with CYPs from the CYP101 Family

**Figure 5:**
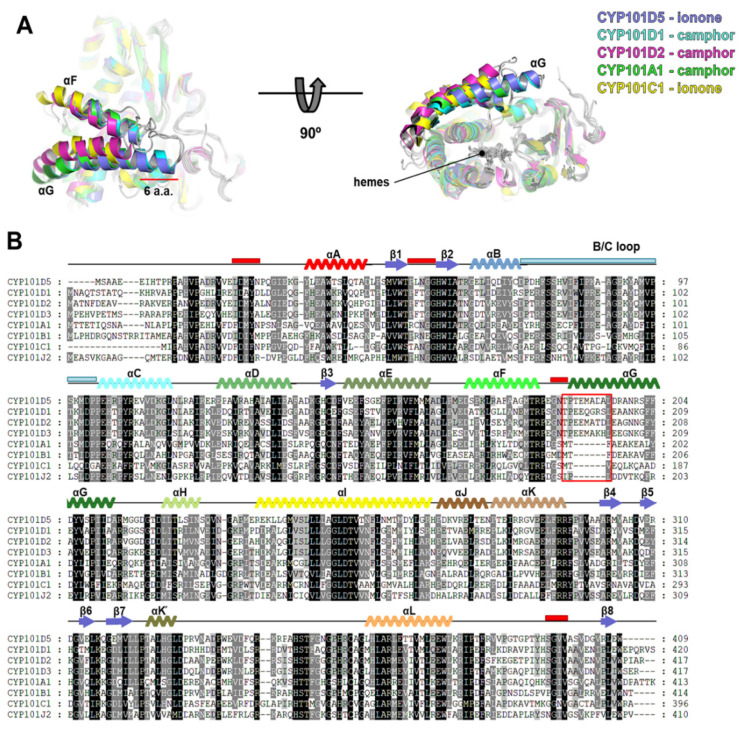
Structural superposition and multiple sequence alignment of CYP101D5 with CYP101 families; CYP101D1, CYP101D2, CYP101B1, and CYP101C1 from *N. aromaticivorans* DSM12444, CYP101D3 from *Sphingomonas* sp. SKA58, CYP101A1 from *P. putida*, and CYP101J2 from *Sphingobium yanoikuyae*. (**A**) Superposition of monomeric structures from CYP101 family highlighting αF, αG, and F/G loop. (**B**) The alignment was performed using ClustalW and visualized using GeneDoc. An additional six amino acids for the CYP101D subfamily are indicated with a red box. Loops co-ordinating the substrate binding site are indicated on the top of the sequences with red and cyan bars. The secondary structures are shown with simple diagrams on the top of the sequences based on the CYP101D5 structure and depicted with multiple colors. The color code for the secondary structure is the same as that of the CYP101D5 structure in Figure 4.

Bacterial CYPs have previously been grouped into CYP families by sequential similarity. Since all of the characterized CYP101 family proteins can catalyze either the hydroxylation of ionone or camphor, we performed pairwise sequence alignment to understand the sequential features for substrate preference and selectivity for the CYP101 family (Figure 5B). As shown in the sequence alignment of the CYP101 family, a significant difference among the CYPs was found in the helix G region. The CYP101D subfamily, which includes D1, D2, D3, and D5, had six additional amino acids compared to CYP101A1, CYP101B1, CYP101C1, and CYP101J1. A structural comparison indicated that the additional amino acids generated the long αG that covered the entrance of the substrate path (Figure 5A). Helix G is located at the top of the CYP active site and has shown a significant shift upon substrate binding in the CYP101 family [20,47,48]. Given that the F/G and B/C loops of CYP101D5 are thought to be essential regions for substrate access with high fluctuation (Figure 4B,C), we assumed that αG is solely responsible for substrate selectivity [18]. However, although these proteins are grouped in the CYP101 family and αG is closely located in the substrate entrance to the active site with low sequential conservation (Figure 4 and Figure S7), to adapt to various substrates, the length of αG may not be a critical factor for substrate selectivity, as CYP101D5 and CYP101C1 use ionones as substrates, whereas CYP101A1, D1, and D2 prefer camphor (Table 3).

### 2.6. Structural Characteristics for Substrate Specificity

**Figure 6:**
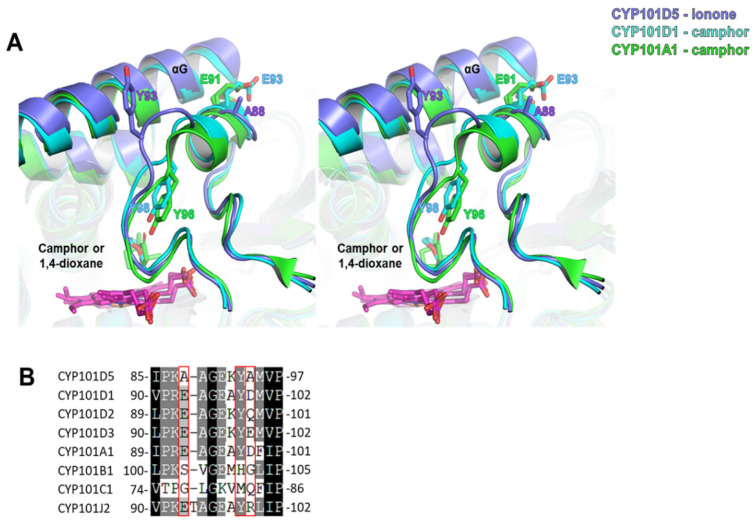
Structural and sequence comparison of the B/C region. (**A**) Stereo view of the CYP101D5 structure overlaid with structurally similar CYP101D1 (PDB: 3LXH) and CYP101A1 (PDB: 4KKY). αF, αG, and the B/C loop are shown with sticks, and the 1,4-dioxane from CYP101D1 and camphor from CYP101A1 are indicated with sticks at the substrate binding site. (**B**) Sequence alignment of the B/C loop region and amino acids. Residues corresponding with the Tyr93 of CYP101D5 and small residues on the B/C loop, which are specific to CYPs with substrate preference for ionone, are marked with red boxes.

A homology search by the DALI server [49] revealed that the most similar structure was CYP101D1 (PDB entry 3LXH) from *N. aromaticivorans* DSM12444, with a Z-score of 59.1 and RMSD of approximately 0.8 Å for approximately 400 aligned residues. The second most similar structure was CYP101A1 (P450cam, PDB entry 4KKY) from *P. putida*, with a Z-score of 54.2. Since these two enzymes have specific activity for camphor, unlike CYP101D5 (Table 4) [50,51,52,53,54], a structural comparison of CYP101D5 with these structures was conducted. The comparison revealed a different conformation in the B/C loop region. CYP101D1 and CYP101A1 form an additional short helix in the B/C loop region and interact with αG. The edge of the additional helix is bent into the active site, and Tyr98 of CYP101D1 and Tyr96 of CYP101A1 form a hydrophilic interaction with the carbonyl group of camphor [55]. Therefore, this interaction is important for the orientation and specificity of camphor [56,57,58]. However, in the structure of CYP101D5, the corresponding loop remained toward the solvent area, and Tyr93 also protruded outwards (Figure 6A). We speculated that small amino acids, such as Ala88 and Ala94, are the points that disturb the formation of the helix and cause different conformations. Similarly, CYP101B1 and CYP101C1, which have a preference for ionone, had no tyrosine or small amino acids in the corresponding regions (Figure 6B). This analysis, therefore, indicates that the different orientations of the B/C loop region, including Tyr96, likely differentiate the substrate preference of the CYP101 family. These findings further explain why CYP101D5 catalyzes the hydroxylation of ionone and not camphor.

**Figure 7:**
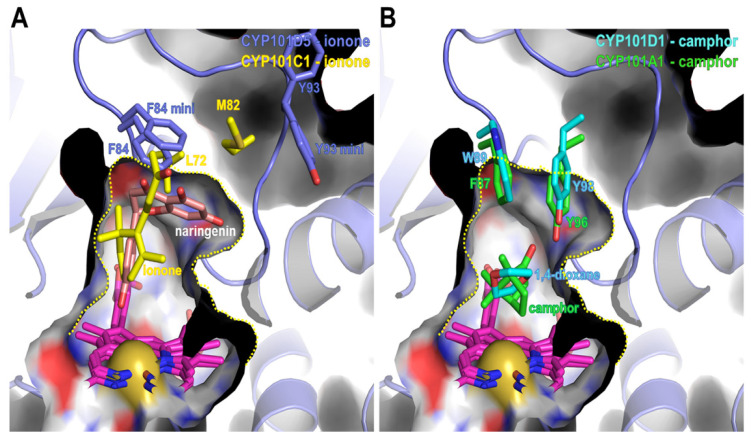
Substrate binding site of CYP101D5 and a comparison of residues consisting of the binding sites of three CYPs from the CYP101 family. The substrate binding site is denoted with a yellow dotted line. (**A**) Ionone-preferring CYPs; CYP101D5 and CYP101C1 are superposed. (**B**) Camphor-binding CYPs are aligned with the CYP101D5 structure. Key residues at the substrate binding site are highlighted with sticks. Residues from the energy-minimized structure of CYP101D5 are indicated and compared with the crystal structure.

It is evident that the CYP101 family accepts small substrates. This is indicative of a narrow active site. However, CYP101D5 also exhibited the bioconversion of relatively large molecules, such as the flavonoids naringenin and apigenin, which are bigger than ionone. To understand this characteristic, we modeled naringenin at the active site of CYP101D5 by superimposition of CYP101C1 complexed with β-ionone (PDB: 3OFU), performed energy minimization, and compared the results with the CYP101 family. The modeled naringenin was located in the upper region of the heme molecule and overlapped with the β-ionone from the CYP101C1 structure. Moreover, the 3′ carbon of the benzene ring connected to the C-2 of an oxygen-containing pyran ring and was located near the center of the heme molecule for 3′ hydroxylation. This indicates that the position of the model in CYP101D5 is reliable (Figure 7).

A structural comparison indicated that CYP101D5 has a larger space at the active site. The active site of camphor binding enzymes, including CYP101D1 and CYP101A1, are surrounded by bulky residues, such as Trp89, Tyr98, and Met100 in CYP101D1 or Phe87, Tyr96, and Phe98 in CYP101A1. These residues appear to be stabilized by hydrophobic interactions and form a small substrate binding site that is oriented toward the active site (Figure S8). In contrast, CYP101C1 and CYP101D5 have relatively small residues at the corresponding locations. The bent loop of the B/C loop mentioned earlier generates additional space for the substrate in CYP101D5. This conformation of the loop with the outward-pointing tyrosine increases the volume of the active site and enables the hydroxylation of larger substrates. Previous studies on CYP101A1 have also stated that the Y96A mutation changes the substrate preference for hydrophobic and larger compounds [57]. The modeling of the naringenin complex structure further indicated that this space could be occupied by naringenin. The two adjacent rings of naringenin were located in the area and interacted with Phe84 with an edge-to-face conformation. This analysis implies that additional space allocation in the active site caused by the turn region of the B/C loop may be a steric determinant for the broad spectrum of substrate acceptance and specificity of CYP101D5.

**Figure 8:**
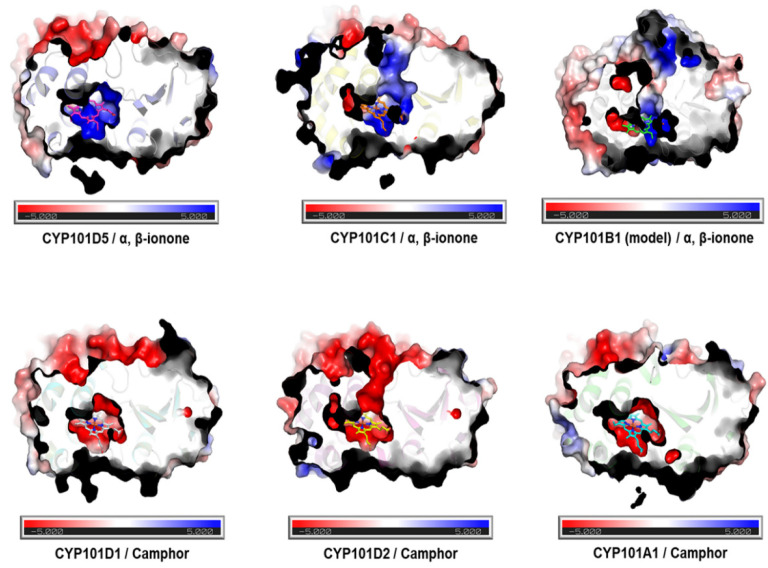
Surface charge distribution of the CYP101 family members. Red represents negatively charged regions (scale of −5), blue represents positively charged regions (scale of +5), and gray represents neutrally charged regions. The ionone-binding CYPs show the distribution of positive charges, especially surrounding the heme molecule and substrate binding site, while camphor-binding CYPs are mostly negative at the substrate binding site, including the substrate path.

Another distinguishable difference was the charge distribution on the substrate binding site of the CYPs, depending on the substrate. A surface charge distribution analysis of the CYP101 family using the Adaptive Poisson–Boltzmann Solver (APBS) revealed that the substrate binding site of the ionone-binding CYPs consisted of positively charged residues (blue). This electrostatic feature was also found in the modeled CYP101B1, which interacted with ionone as a substrate. In contrast, negatively charged residues (red) were locally distributed in the adjacent regions of the substrate binding site in the camphor-binding CYPs (Figure 8). The same charge distribution trait was also found at the substrate path of CYP101B1, C1, and D2. Since only positively charged CYPs at the active site bind to the ionones, while negatively charged CYPs bind to camphor, the electric field may be one of the factors involved in substrate selectivity and recognition [60].

To date, CYP101 family proteins have been renowned for the hydroxylation of small molecules, such as camphor and ionones. Camphor oxidation is catalyzed by CYP101A1, CYP101D1, and CYP101D2, while CYP101B1 and CYP101C1 use ionone. However, CYP101D5 showed unusual features on both the catalysis and the substrate. The dehydrogenation of ionone is an entirely novel reaction in the CYP101D subfamily. Furthermore, CYP101D5 demonstrated the hydroxylation of a larger substrate, such as flavonoid, which has not been observed in the CYP101D family and is the rarely occurring biotransformation catalyzed by bacterial CYPs. The structural comparison between CYP101D5 and other CYP101 families indicates that alternation of the B/C loop’s orientation generating the larger active site and charge distribution on the substrate binding site are key points changing the substrate preference of CYP101D5. Although previous studies showed that the B/C loop might be responsible for the small substrate recognition, a different orientation of the B/C loop for large substrate has not been shown in structures of the CYP101 family. Therefore, our biochemical and structural results and data will provide the basic information or rationale for changing the substrate preference and can be used as a starting model for the structure-based protein engineering of the CYP101 family.

## 3. Materials and Methods

### 3.1. Chemicals and Enzymes

Alpha- and beta-ionone, steroids, and monoterpenoids were purchased from Tokyo Chemical Industry Co., Ltd. (Seoul, Korea). All of the flavonoids used in this study, δ-aminolevulinic acid (ALA), formate dehydrogenase, ampicillin, and NADH were purchased from Sigma-Aldrich (Yongin, Korea). Isopropyl-1-thio-β-D-galactopyranoside (IPTG), 1, 4-dithiothreitol (DTT), and kanamycin were purchased from Duchefa Biochemie (Haarlem, The Netherlands)). Restriction enzymes, T4 DNA ligase, dNTPs, and DNA polymerase were purchased from Takara Bio (Shiga, Japan). All other high-grade chemicals were purchased from available commercial sources.

### 3.2. Sequence Accession Number

The CYP gene was searched for in *S. echinoides* based on the signature heme-binding domain (FXXGX(H/R)XCXG). The name of the enzyme (CYP101D5) was assigned by Dr. David Nelson [10]. The nucleotide sequences of CYP101D5 have been deposited in GenBank under the accession number ON416863.

### 3.3. Cloning, Overexpression, and Purification of CYP101D5

Oligonucleotide primers (Geno-Tech, Korea), including 5′-GAA TTC ATG AGC GCC GCC GAA GAG-3′ (*Eco*RI site underlined) as the forward primer and 5′-AAG CTT TCT AGC CGG TCA CCA TTC CA-3′ (*Hin*dIII site underlined) as the reverse primer, were designed. The target gene was amplified and cloned into the pET28a(+) vector. Under the control of the IPTG-induced T7 phage promoter and with an N-terminal His6-tag, the DNA construct was introduced into *E. coli* BL21 (DE3) cells. For protein expression, the transformed cells were grown overnight at 37 °C for seed culture and inoculated into LB medium with 50 µg/mL of kanamycin. When the cell density reached 0.6 at OD_600_, the culture was supplemented with 1.0 mM ALA and 0.5 mM FeCl_3_, followed by induction with 1.0 mM IPTG. The cells were incubated for 48 h at 20 °C to assess protein expression. The cell pellets were collected and washed twice with 50 mM phosphate buffer (pH 7.4) containing 10% glycerol. For purification, the cells were homogenized, and the soluble protein fraction was separated after centrifugation. The soluble fraction was mixed with pre-equilibrated TALON His-tag resin by equilibrium buffer (potassium phosphate buffer, pH 7.4). Resin-bound proteins were eluted with elution buffer (potassium phosphate buffer, pH 7.4, with 10% glycerol) containing 10 mM, 100 mM, and 250 mM imidazole, respectively. The fractions containing proteins were concentrated by ultrafiltration using Amicon centrifugal filters (Millipore) with a molecular mass cutoff of 30 kDa. The protein obtained was checked by 15% SDS-PAGE. For the electron transport system, the overexpression and purification of Pdx and PdR were conducted based on a previously published protocol [61].

The concentration of CYP101D5 was estimated based on the CO difference spectra using the extinction coefficient ε_450-490_ = 91 mm^−1^ cm^−1^ [62,63]. The protein was diluted with potassium phosphate buffer and separated into two cuvettes (reference and sample), each containing 1 mL of the sample. The spectrum was recorded using the Biochrome Libra S35PC UV/Visible Spectrophotometer (Cambridge, UK) after bubbling carbon monoxide gas to the sample cuvette at a rate of 1 bubble per second for 1 min and reducing both the reference and sample by adding a few grains of sodium dithionite. The concentration of PdR was determined based on the average concentration calculated from wavelengths of 378 nm, 454 nm, and 480 nm using the extinction coefficient (ε) = 9.7, 10.0, and 8.5 mM^−1^cm^−1^, respectively [64]. The concentration of Pdx was determined using the extinction coefficient (ε) = 11.1 and 10.4 mM^−1^cm^−1^ at wavelengths of 415 nm and 454 nm, respectively [61].

### 3.4. Enzyme Activity Assay

The in vitro activity of CYP101D5 was determined using the redox partner Pdx/PdR in 50 mM potassium phosphate buffer (pH 7.4). All the substrates were prepared by being dissolved in dimethyl sulfoxide (DMSO). The reaction mixture contained CYP (3 µM), substrate (100 µM), PdR (6 µM), Pdx (24 µM), catalase (100 µg/mL), and an NADH regeneration system comprising formate dehydrogenase (1 U), sodium formate (150 mM), and MgCl_2_ (1 mM) in phosphate buffer (pH 7.4). The reaction was initiated by 250 µM NADH, followed by incubation for 2 h at 30 °C with shaking. The reaction mixture was extracted with a double volume of ethyl acetate, which was dried, dissolved in methanol, and analyzed by an HPLC-PDA and LC–MS or GC–MS.

### 3.5. Kinetics Analysis

An enzyme kinetics study was performed in the reaction system consisting of CYP101D5 (1 µm), PDX (8 µM), PDR (2 µM), and 500 µM NADH in phosphate buffer. The time-dependent reaction progress curve was first generated by measuring the amount of product formed over time using substrates. The initial velocity condition was then established, and the saturation curve was generated using a varied substrate concentration of 0–400 μM. The kinetic parameters were calculated from the plot of the reaction rate versus the substrate concentration. Coupling efficiency was determined as the percentage of NADH utilized for product formation over the total consumption of NADH [65]. The kinetics analysis was performed using a non-linear regression analysis based on Michaelis–Menten kinetics using the OriginPro program (OriginLab Corporation, Northampton, MA, USA).

### 3.6. Whole-Cell Bioconversion

Whole-cell bioconversion was performed in *E. coli* cells harboring genes for CYP101D5, PdR, and Pdx. The cells were grown with the appropriate antibiotics at 37 °C. The culture was supplemented with 1 mM ALA and 0.5 mM FeCl_3_ and induced by a final concentration of 0.5 mM IPTG when the OD_600_ reached 0.6, followed by incubation for 48 h at 20 °C. The cells were collected, washed twice with phosphate buffer (pH 7.4), and resuspended in the same buffer supplemented with 1.0 mg/mL of glucose and 1.0 mM of the substrate. Bioconversion was performed for 24 h at 30 °C. The sample was then extracted twice with an equal volume of ethyl acetate, dried, and analyzed.

### 3.7. Analytical Methods

The dried ethyl acetate fraction collected from the in vitro and in vivo reactions was dissolved in HPLC-grade methanol, filtered, and analyzed by HPLC-PDA using a reversed-phase column (Mightysil RP–18 GP 250–x4.6 I.D., 5 µm, Kanto Chemical, Tokyo, Japan). Separation was achieved using gradient mobile phase composed of solvent A (0.05% trifluoroacetic acid in HPLC-grade water) and solvent B (100% acetonitrile, CH_3_CN). The percentage of solvent B used was as follows: 10% (0 to 5 min), 50% (5 to 10 min), 70% (10 to 14 min), 90% (14 to 17 min), 10% (17 to 20 min), and 10% (20 to 25 min), with a flow rate of 1.0 mL/min. The oven temperature was set to 40°C, and the detection of the substrate and its products was performed by UV absorbance at their respective wavelengths. An LC–MS analysis of the products was performed by HR-QTOF ESI/MS in positive ion mode using an ACQUITY (UPLC, Waters Corp., Billerica, MA, USA) column coupled with an SYNAPT G2-S (Water Corp.). The products were purified using preparative HPLC (Shimadzu, Tokyo, Japan) with a C18 column (YMC–Pack ODS-AQ (150 × 20 mm I.D., 10 µm), UV detector, and with a 35 min binary program with different concentrations of acetonitrile: 15% (0 to 3 min), 25% (3 to 7 min), 40% (7 to 12 min), 45% (12 to 15 min), 50% (15 to 17 min), 90% (17 to 23 min), 90% (23 to 25 min), 10% (25 to 28 min), and 10% (28 to 35 min) at a flow rate of 10 mL/min.

GC–MS analyses were performed using an Agilent 5977B GC/MSD. One microliter of each sample was injected using an autosampler with a split ratio of 1:10. Separation was performed on an Rtx-5MS capillary column (30 m × 0.25 mm × 0.25 μm) using helium as the carrier gas with a flow rate of 1.0 mL/min. The initial temperature was maintained at 40 °C for 5 min, then increased to 300 °C at 10 °C min^−1^. This temperature was maintained for 5 min. During the identification process, the mass spectra of the compound were compared with the mass spectral data available in the NIST12 library.

The purity of the hydroxylated products was reconfirmed by HPLC. For structure elucidation, fractions containing the purified hydroxylated products were dried, lyophilized, and dissolved in DMSO-*d*_6_. Finally, the sample was subjected to NMR analyses at 700 MHz by Bruker Biospin GmbH (Rheinstetten, Germany). The NMR spectra were analyzed to determine the structure using MestReNOVA version 14.0.1.

Eriodictyol: ^1^H NMR (700 MHz, DMSO-*d*_6_) δ 12.15 (s, 1H), 6.88 (s, 1H), 6.76–6.74 (m, 2H), 5.89–5.88 (m, 1H), 5.38 (dd, J = 12.5, 3.1 Hz, 1H), 3.19 (dd, J = 17.2, 12.5 Hz, 1H), 2.68 (dd, J = 17.1, 3.2 Hz, 1H); ^13^C NMR (176 MHz, DMSO-*d*_6_) δ 196.33 (C4), 166.77 (C7), 163.50 (C5), 162.92 (C9), 145.72 (C4′), 145.20 (C3′), 129.48 (C1′), 117.96 (C6′), 115.35 (C5′), 114.35 (C2′), 101.77 (C10), 95.79 (C6), 95.00 (C8), 78.46 (C2), 42.09 (C3). The NMR spectral values were identical to data published in the literature [30,31].

Luteolin: ^1^H NMR (700 MHz, DMSO-*d*_6_) δ 12.98 (s, 1H), 7.42 (dd, J = 8.2, 2.2 Hz, 1H), 7.40 (d, J = 2.4 Hz, 1H), 6.90 (d, J = 8.3 Hz, 1H), 6.67 (s, 1H), 6.45 (d, J = 2.2 Hz, 1H), 6.19 (d, J = 2.1 Hz, 1H); ^13^C NMR (176 MHz, DMSO-*d*_6_) δ 181.67 (C4), 164.17 (C2), 163.90 (C7), 161.49 (C5), 157.30 (C9), 149.72 (C4′), 145.75 (C3′), 121.51 (C1′), 119.00 (C6′), 116.03 (C5′), 113.38 (C2′), 103.70 (C10), 102.87 (C3), 98.85 (C6), 93.86 (C8). The NMR spectral values were identical to data published in the literature [32].

### 3.8. Crystallization, Data Collection, and Structure Determination

Crystals were obtained by the sitting-drop vapor diffusion method at 22 °C by mixing an equal volume of protein solution (50 mg/mL of CYP101D5) and the precipitant solution containing 1.6 M ammonium phosphate monobasic. Before the X-ray diffraction test, the crystals were transferred to a cryoprotectant solution consisting of the precipitant solution with 20% glycerol and incubated for 1 min. Diffraction data were collected on the beamline 5C at the Pohang Accelerator Laboratory, Korea, and processed using HKL 2000 [66]. The initial phase of the co-ordinate was obtained by molecular replacement using the program MOLREP from the CCP4i suite [67], using CYP101D2 from *N. aromaticivorans* DSM 12444 as a search model [54]. Coot [39], refmac5 [40], and PHENIX [68] were used to build the model. The figures were generated using PyMOL [69].

### 3.9. Modeling of CYP101B1

Since there were no available structures for CYP101B1, the (Iterative Threading ASSEmbly Refinement (I-TASSER) structure prediction server was utilized to model CYP101B1 [54,70]. The multiple threading alignments and iterative structural assembly simulations generated the CTP101B1 model with 0.53 and 0.78 ± 0.53 of the confidence score (c-score) and template modeling score (TM-score), respectively [70].

### 3.10. Substrate Channel Prediction of CYP101D5

The substrate channels in CYP101D5 were analyzed using the CAVER software in PyMOL [69]. The structure of CYP101D5 without water molecules was used, and the starting point was set to the heme molecules. The minimum probe radius, shell depth, shell radius, and clustering threshold were 0.9, 4, 3, and 3.5, respectively.

### 3.11. Amino Acid Conservation Analysis

Amino acid conservation in CYP was estimated using the ConSurf server [71]. The monomeric structure of CYP101D5 was used to search for homologues using the PSI-BLAST search algorithm. The blasted sequences were analyzed using the maximum likelihood (ML) approach. A total of 400 homologues were analyzed for the conservation scoring.

## 4. Conclusions

We characterized the newly identified CYP101D5 from *S. echinoides* and described its structure, which contains heme as a cofactor. A biochemical study showed that CYP101D5 possesses enzymatic activity for a wide range of substrates. An initial activity measurement of CYP101D5 with substrate candidates revealed that CYP101D5 catalyzes the hydroxylation of β-ionone and flavonoids, such as naringenin and apigenin, and the dehydrogenation of α-ionone. Therefore, CYP101D5 produces the 3′-positioned 4-hydroxy β-ionone, 3′,4′,5,7-tetrahydroxyflavanone (eriodictyol), and 5,7,3′,4′-tetrahydroxyflavone (luteolin). We also observed the unusual dehydrogenation activity of CYP101D5 on α-ionone.

A comparative analysis of CYP101D5 with members of the CYP101 family revealed that CYP101D5 could be superposed on other enzymes from the family to a high degree. However, CYP101D5 has a unique conformation at the substrate binding site that is influenced by the edge region of the B/C loop. Short residues and the orientation of Tyr93 appear to form favorable conformations for larger substrates. Thus, the spatial constraints at the substrate recognition site and charge distribution at the substrate binding site may be important factors for the substrate selectivity and preference of these proteins. Although the mechanisms underlying the dehydrogenation of α-ionone by CYP101D5 remain to be fully elucidated, our structural analysis and biochemical investigation of CYP101D5 provide insights into the B/C loop of bacterial CYPs that play an essential role in biocatalysis.

## Figures and Tables

**Table 1:** Michaelis–Menten constant (*Km*), catalytic rate constant (*k_cat_*), and coupling of naringenin and apigenin by CYP101D5. The calculation of the kinetic parameters was performed using a CYP:Pdx:PdR ratio of 1:8:2. Coupling efficiency was calculated as the fraction of NADH utilized for product formation over the total consumption of NADH. The background NADH consumption rate was subtracted to calculate the actual NADH consumption rate. The results represent the mean values of triplicate measurements.

Substrate	*K_m_* (μM)	*k_cat_* (min^−1^)	Coupling Efficiency (%)
Naringenin	26.39 ± 2.23	1.17 ± 0.08	39.84 ± 4.39
Apigenin	46.28 ± 4.88	0.83 ± 0.08	36.25 ± 5.08

**Table 2:** X-ray diffraction data collection and refinement statistics.

Data Collection	
Crystal	CYP101D5
X-ray source	BL-5C beam line
Space group	P212121
Unit-cell parameters (Å, °)	a = 68.52., b = 109.57, c = 113.87, α = β = γ = 90.00
Wavelength (Å)	0.9794
Resolution (Å)	40.67–3.20 (3.31–3.20)
Total reflections	402,646
Unique reflections	14,555 (1195)
Average I/σ (I)	10.1 (1.2)
R_merge_^a^	0.082 (0.43)
Redundancy	4.6 (4.5)
Completeness (%)	99.4 (99.2)
**Refinement**	
Resolution range (Å)	40.67–3.20 (3.31–3.20)
No. of reflections	14,506 (1330)
No. of amino acid residues	798
No. of water molecules	13
Molecules per asymmetric unit	2
R_cryst_^b^	0.2607 (0.3741)
R_free_^c^	0.3216 (0.4343)
Rotamer outliers (%)	0.00
R.m.s. bond length (Å)	0.002
R.m.s. bond angle (°)	0.58
**Ramachandran plot**	
Favored (%)	91.92
Allowed (%)	6.57
Outliers (%)	1.52

^a^ R_merge_ = ∑|<I> − I|/∑<I>. ^b^ R_cryst_ = ∑||Fo| − |Fc||/∑|Fo|. ^c^ R_free_ calculated with 5% of all reflections excluded from the refinement stages using high-resolution data. Values in parentheses refer to the highest resolution shells.

**Table 3:** Substrate preference of CYP101 family proteins and the RMSD values compared to CYP101D5.

Protein	Substrate	PDB Code	Cα RMSD	References
CYP101D5	α/β-ionone		0	This study
CYP101D1	camphor	3LXH	0.826	[18]
CYP101D2	camphor	3NV5	1.345	[18]
CYP101A1	camphor	2CPP	1.11	[47]
CYP101B1	β-ionone	No structure available	-	
CYP101C1	β-ionone	3OFU	1.55	[20]

**Table 4:** Structural homologue search results for CYP101D5 from a DALI search (DALI-Lite server).

CYP Annotation/Protein	PDB Code	DALI Z-Score	UniProt/ KB Code	Sequence % ID with CYP101D5 (Aligned Residue Number)	Reference
CYP101D1	3LXH	59.1	Q2GB12	55 (397/408)	[18]
CYP101A1 (P450cam)	4KKY	54.2	P00183	45 (390/411)	PDB deposit only
CYP101C1	3OFT	48.7	Q2G637	37 (380/396)	[20]
CYP101J2	5KYO	45.5	A0A1C9CIU0	37 (380/394)	[50]
P450cin	1T2B	45.3	Q8VQF6	24 (382/397)	[41]
CYP199A2	4DNJ	41.3	Q6N8N2	22 (381/399)	[51]
P450eryF	1EGY	41.2	Q00441	22 (378/403)	[52]
CYP268A2	6BLD	41.2	B2HMF7	23 (380/414)	[53]
MycCI	5FOI	41.1	Q83WF5	20 (373/389)	[54]
SgvP	4MM0	41.1	R9USI6	23 (373/394)	PDB deposit only
OleP	6ZI3	40.6	Q59819	24 (376/403)	[59]

## Data Availability

The co-ordinate and structural factors were deposited in the Protein Data Bank under PDB ID: 8GTL.

## Supplementary Materials

The following supporting information can be downloaded at: https://www.mdpi.com/article/10.3390/ijms232113317/s1.
