# Supplementary material for: Crystal Structure and Biochemical Analysis of a Cytochrome P450 CYP101D5 from Sphingomonas echinoides
Source: Int J Mol Sci. 2022 Nov 1;23(21):13317. doi: 10.3390/ijms232113317 (PMC9655578; doi:10.3390/ijms232113317)

# Supplementary Material
# on

# Crystal structure and biochemical analysis of a cytochrome P450 CYP101D5 from *Sphingomonas echinoides*


**Pradeep Subedi[1,†], Hackwon Do[2,3,†], Jun Hyuck Lee[2,3,*], and Tae-Jin Oh[1,4,5,*]**

[1] Department of Life Science and Biochemical Engineering, Graduate School, Sun Moon University, Asan 31460, Korea

[2] Research Unit of Cryogenic Novel Material, Korea Polar Research Institute, Incheon 21990, Korea

[3] Department of Polar Sciences, University of Science and Technology, Incheon 21990, Korea

[4] Genome-based BioIT Convergence Institute, Asan 31460, Korea

[5] Department of Pharmaceutical Engineering and Biotechnology, Sun Moon University, Asan 31460, Korea

[†]These authors contributed equally to this work.

*** To whom correspondence should be addressed:**

Dr. Jun Hyuck Lee, Unit of Polar Genomics, Korea Polar Research Institute, Incheon 21990, Republic of Korea, Tel.: +82-32-760-5555; Fax: +82-32-760-5509; E-mail: junhyucklee@kopri.re.kr.

Prof. Tae-Jin Oh, Department of Pharmaceutical Engineering and Biotechnology, Sun Moon University, Asan 31460, Republic of Korea, Tel.: +82-41-530-2677; Fax: +82-41-530-2279; E-mail: tjoh3782@sunmoon.ac.kr.



**Keywords:** α/β-ionone; crystal structure; cytochrome P450; X-ray crystallography

**Figure S1.** (A) SDS-PAGE analysis of purified CYP101D5 (lane 1, 45.97 kDa) and the redox partners PxR (lane 2, 58 kDa) and Pdx (lane 4, 10 kDa), where M is the standard protein marker with the size of the protein marker presented in kilodaltons (kDa). (B) Absorbance spectra of the carbon-monoxide-bound and dithionite reduced forms of CYP101D5.

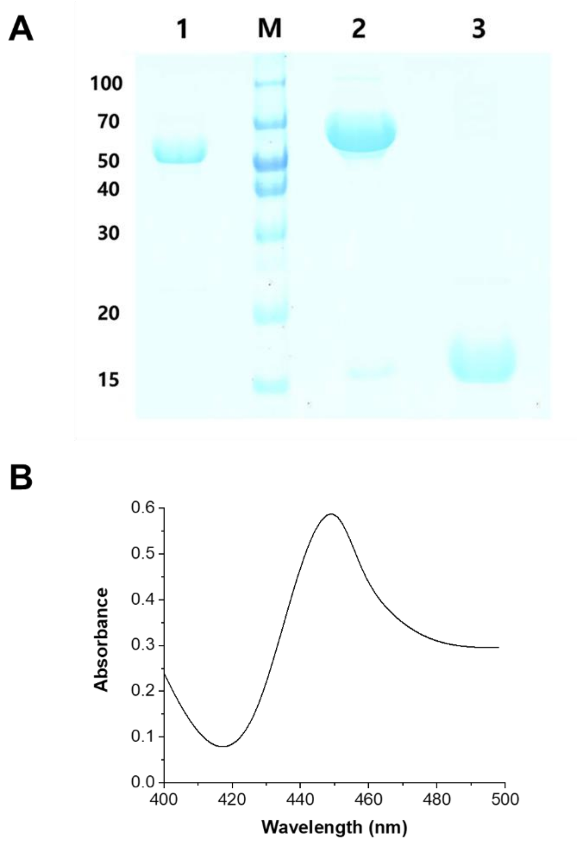

**Figure S2.** Gas chromatogram (GC) spectra of β-ionone conversion by CYP101D5. GC of (A) the control reaction and (B) reaction mixture. The peak ($t_{RS}$ = 13.028 min) is of β-ionone, and the product peak ($t_{RP}$ = 14.134 min) was identified as 4-hydroxy-β-ionone. The structures of both the substrate and product are shown. (C) (D) The mass spectra of the peak and library hits for both the substrate and product are presented.

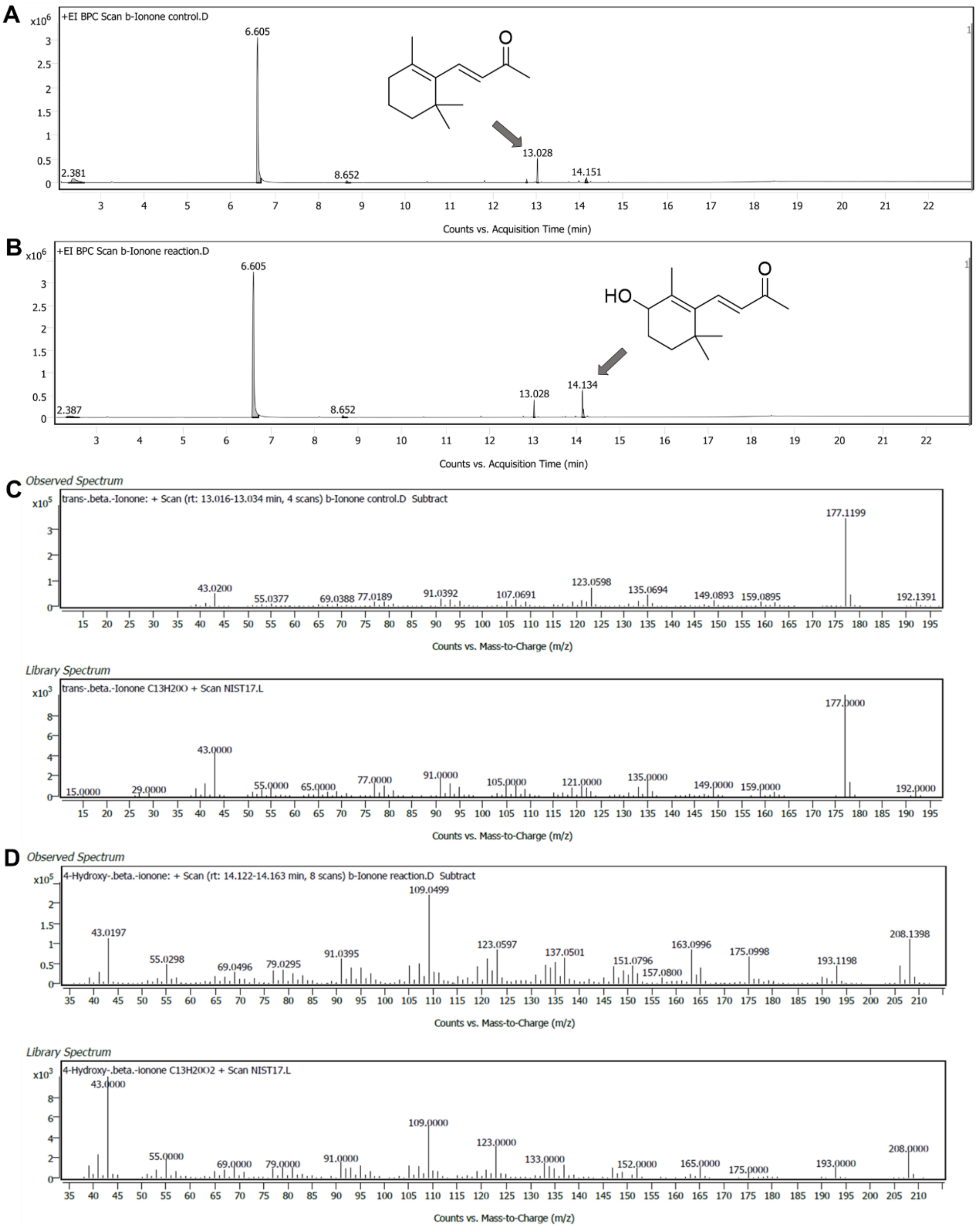

**Figure S3.** MS spectra of both the substrate (α-ionone) (A) and product (2,3-dehydro-α-ionone) (B) peaks. The observed mass spectrum of the peak and library spectrum for both the substrate and product are presented.

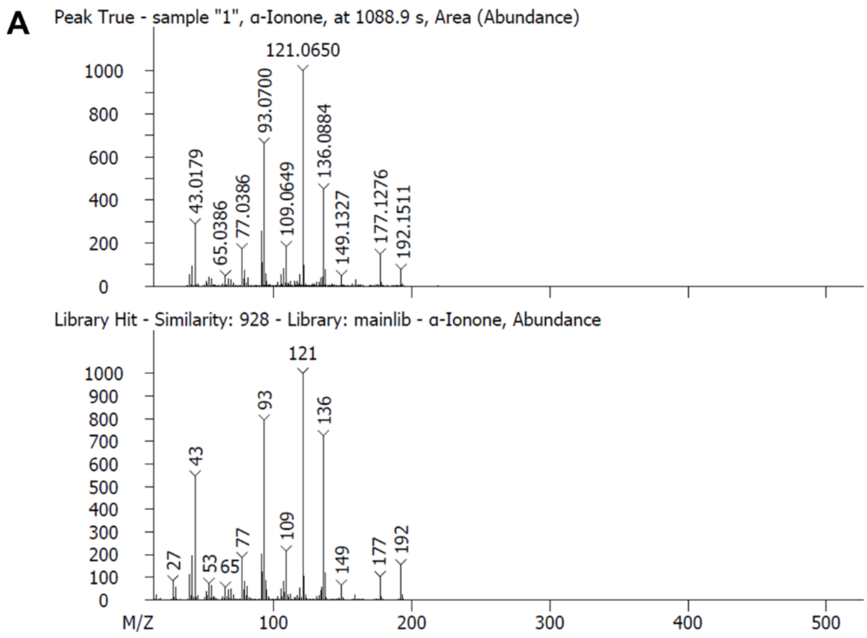


| Peak # | Name | R.T. (s) | Mass Accuracy (ppm) | Similarity | Observed Ion m/z | Expected Ion m/z |
|---|---|---|---|---|---|---|
| 2 | α-Ionone | 1088.9 | 1.32 | 928 | 192.1511 | 192.1509 |

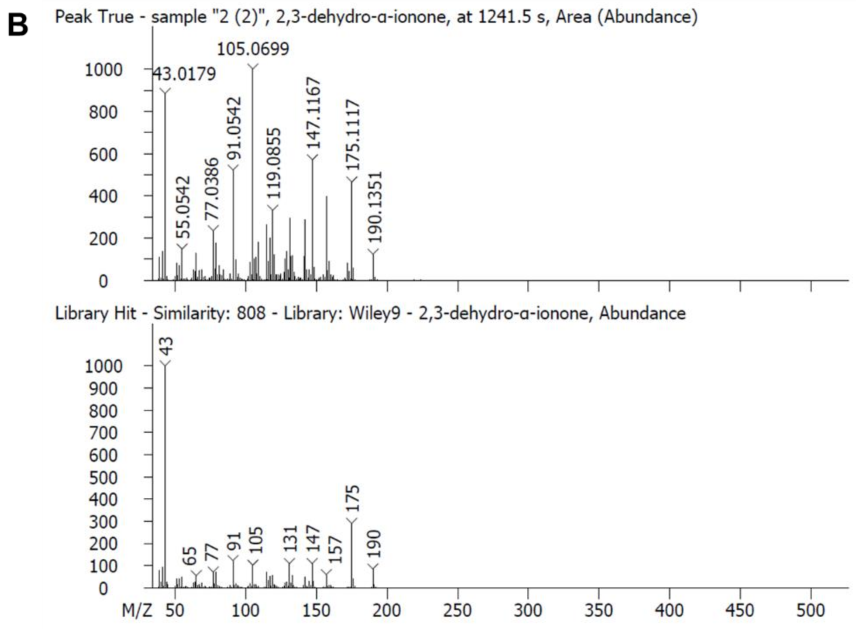


| Peak # | Name | R.T. (s) | Mass Accuracy (ppm) | Similarity | Observed Ion m/z | Expected Ion m/z |
|---|---|---|---|---|---|---|
| 2 | 2,3-dehydro-α-ionone | 1241.5 | -0.71 | 808 | 190.1351 | 190.1352 |

**Figure S4.** HPLC-PDA and HR-QTOF ESI/MS analyses of the reaction products. The product of naringenin (A) and apigenin (B) is denoted by P in their respective HPLC chromatograms. The mass spectra of the substrate and their corresponding hydroxylated product are also shown.

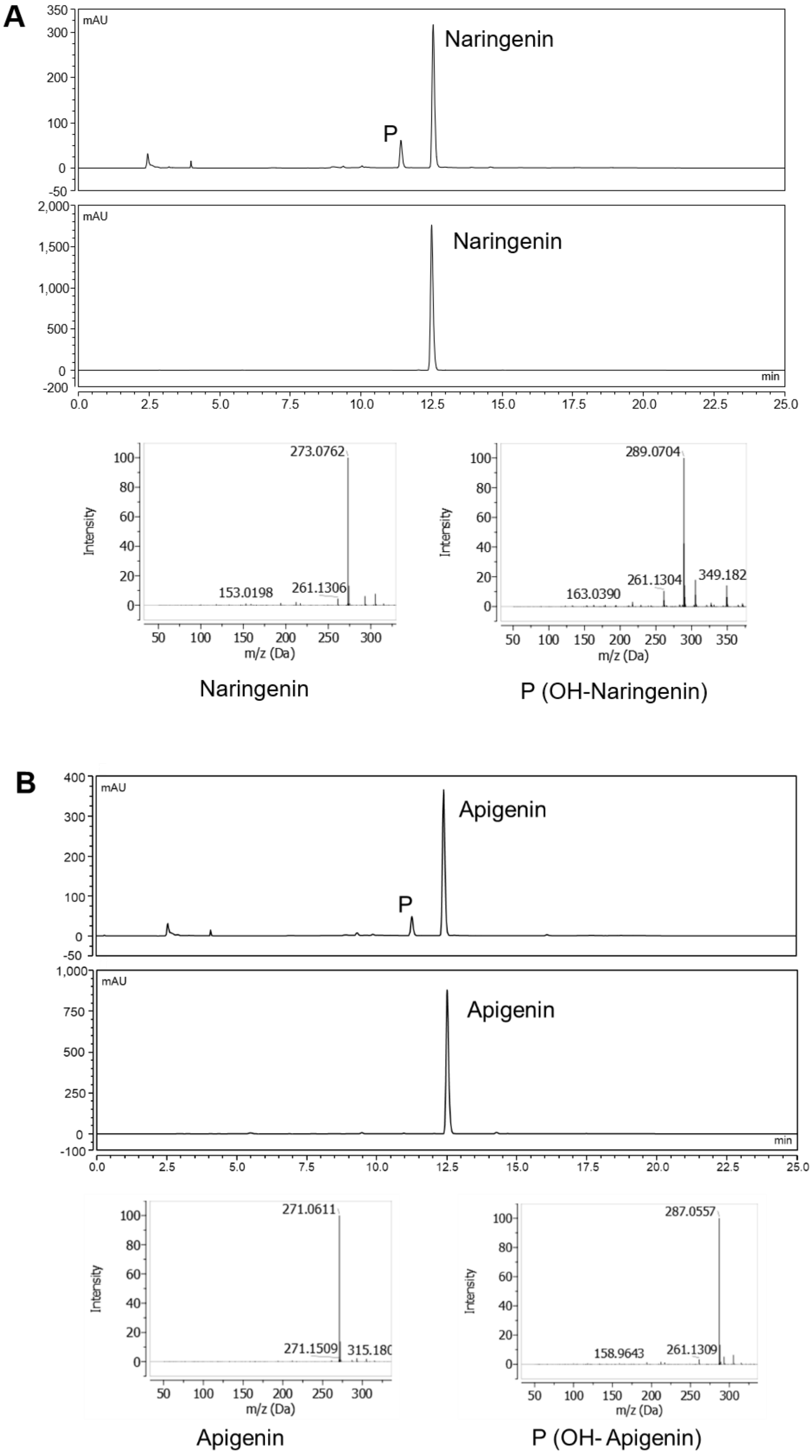

**Figure S5.** $^{1}$H and $^{13}$C NMR analyses of the hydroxylated product of naringenin (A) and apigenin (B).

**A**

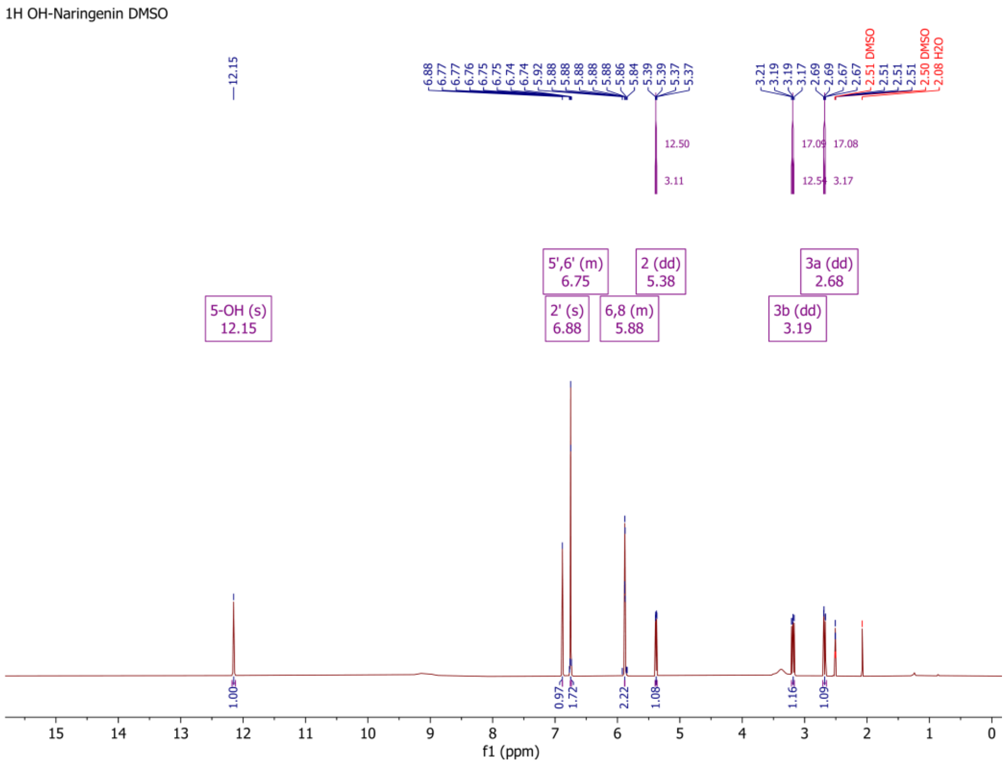


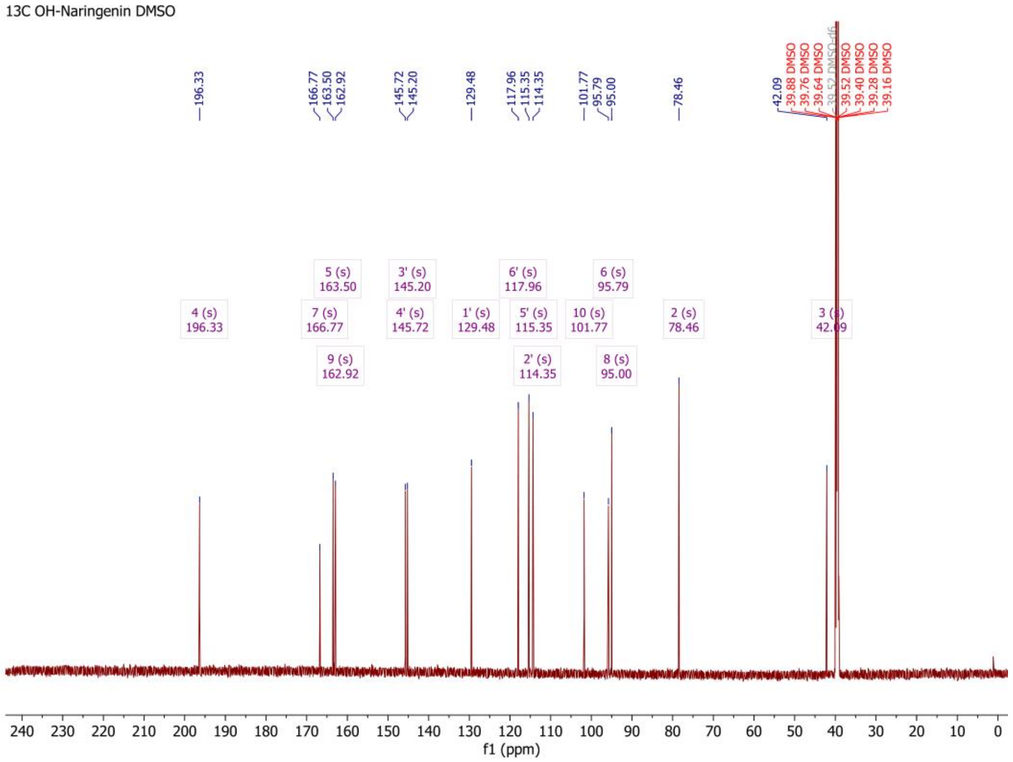

**B**

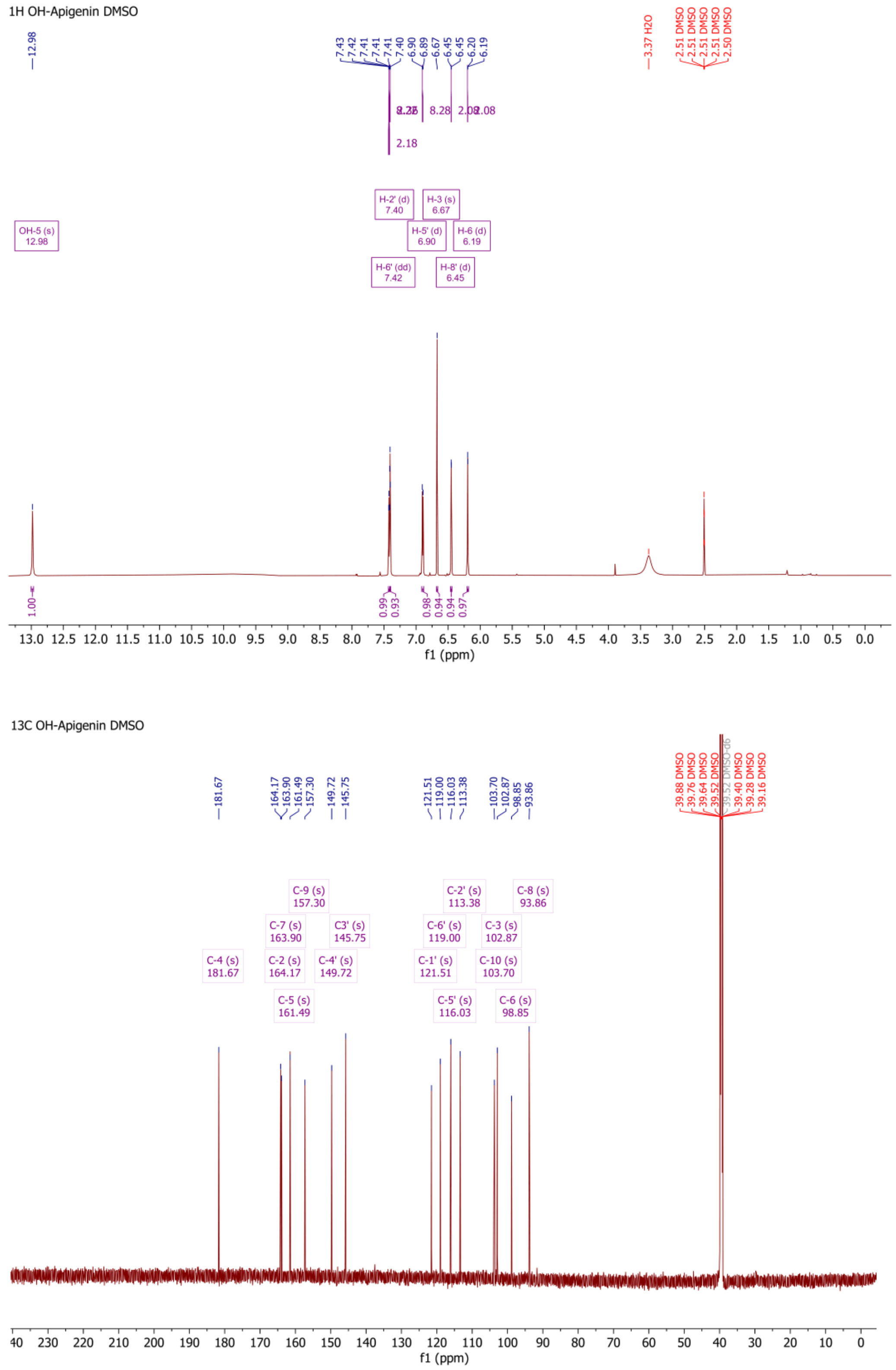
1H OH-Apigenin DMSO
12.98
7.43 7.42 7.41 7.41 7.41 7.40 6.90 6.89 6.67 6.45 6.45 6.20 6.19
3.37 H2O
2.51 DMSO 2.51 DMSO 2.51 DMSO 2.51 DMSO 2.50 DMSO
OH-5 (s) 12.98
H-2' (d) 7.40
H-3 (s) 6.67
H-5' (d) 6.90
H-6 (d) 6.19
H-6' (dd) 7.42
H-8' (d) 6.45
1.00
0.99 0.93
0.98 0.94 0.94 0.97
f1 (ppm)
13C OH-Apigenin DMSO
181.67
164.17 163.90 161.49 157.30
149.72 145.75
121.51 119.00 116.03 113.38
103.70 102.87 98.85 93.86
39.88 DMSO 39.76 DMSO 39.64 DMSO 39.52 DMSO 39.52 DMSO-d6 39.40 DMSO 39.28 DMSO 39.16 DMSO
C-9 (s) 157.30
C-2' (s) 113.38
C-8 (s) 93.86
C-7 (s) 163.90
C3' (s) 145.75
C-6' (s) 119.00
C-3 (s) 102.87
C-4 (s) 181.67
C-2 (s) 164.17
C-4' (s) 149.72
C-1' (s) 121.51
C-10 (s) 103.70
C-5 (s) 161.49
C-5' (s) 116.03
C-6 (s) 98.85
f1 (ppm)

**Figure S6.** The αK′–αL loops are mainly involved in the interaction with the heme molecule through a combination of hydrophobic and hydrogen bonds and salt bridges.

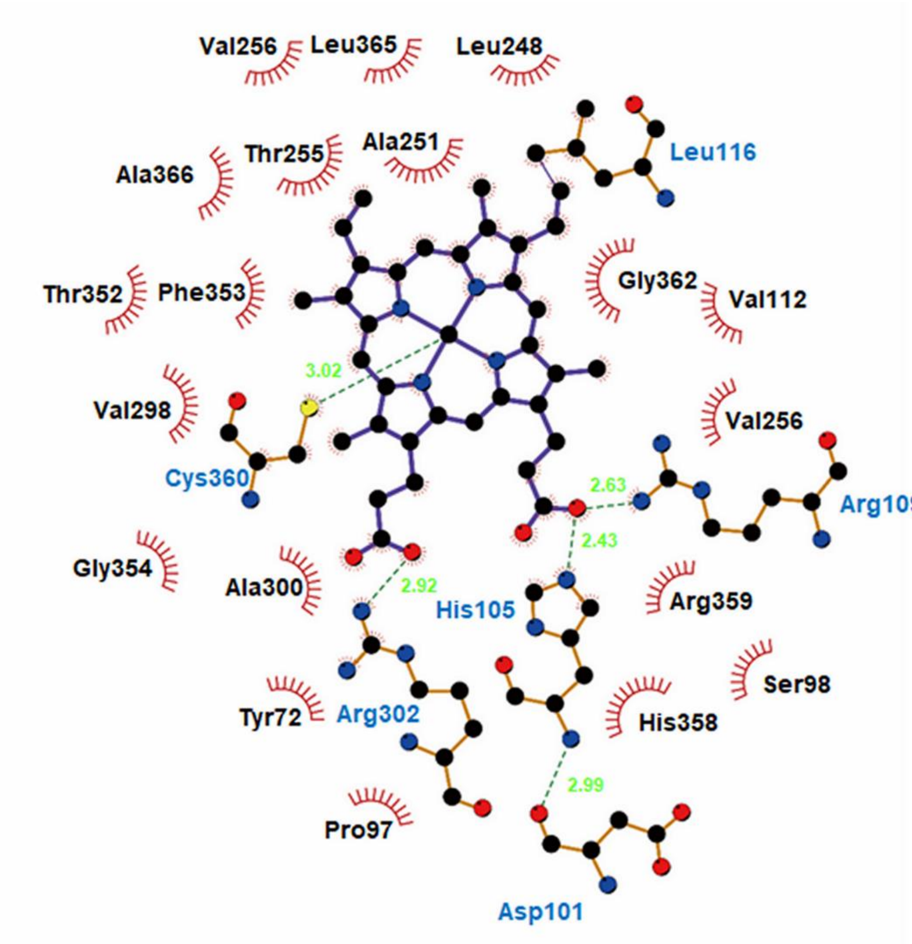

**Figure S7.** Conservation analysis of the protein sequence of CYP101D5 using ConSurf [35]. The conservation of the amino acids among the 400 CYP homologues are indicated using nine-grade color coding. The average scores of the edge B/C and F/G loops were 4.3 and 3.6, respectively.

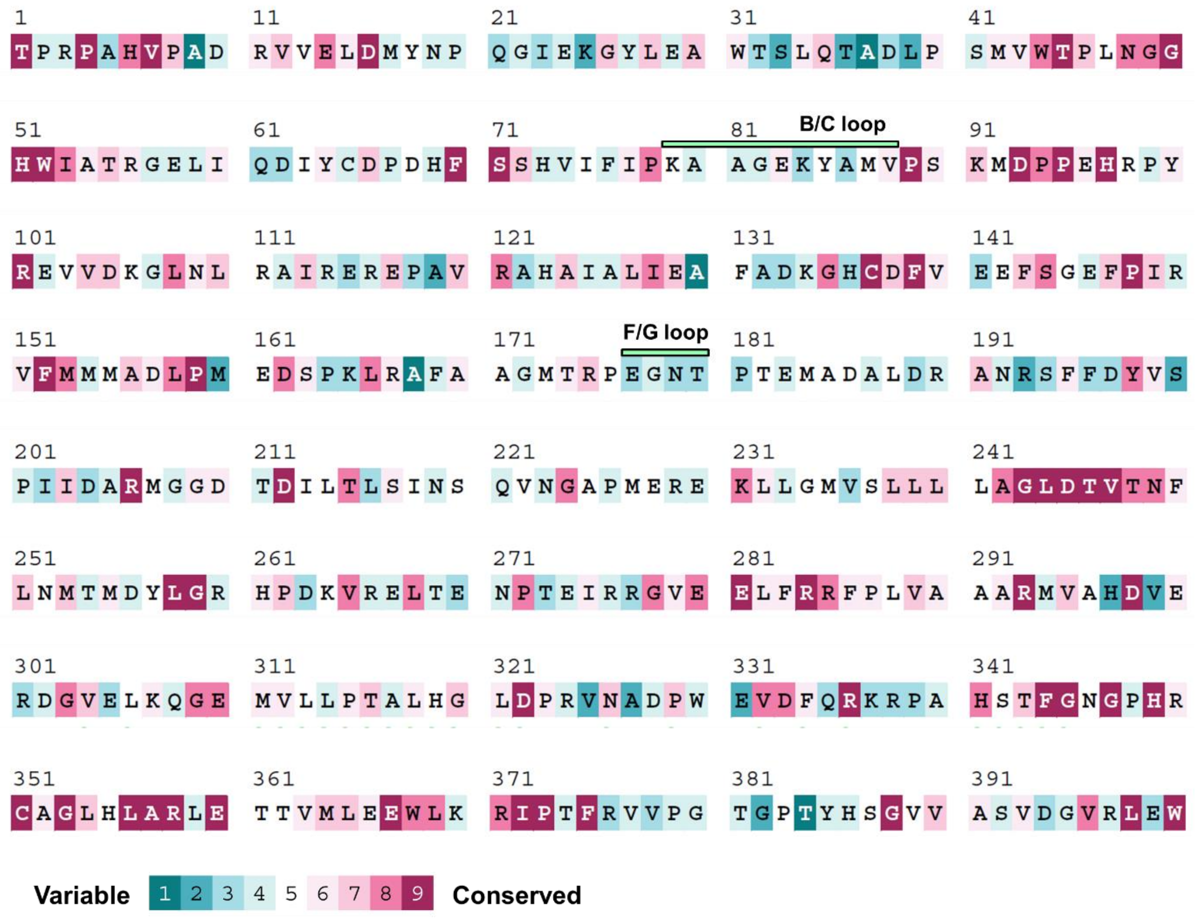

**Figure S8.** Substrate-dependent conformation of the B/C loop in the CYP101 family. Ionone-binding CYPs (A) and camphor-binding CYPs (B) were grouped and presented with different colors. The residues from the B/C loop comprising substrate binding sites are shown with sticks.

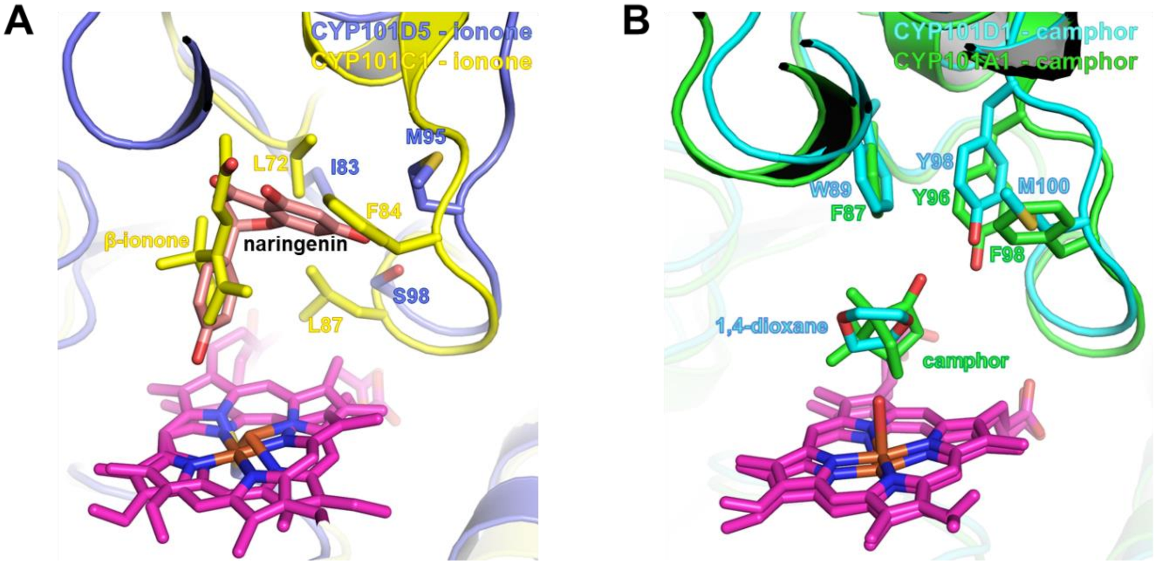

Supplement: Supplementary file 1 [file ijms-23-13317-s001.zip › ijms-1976238-supplementary.pdf]
